# An Emerging Anti-p16 Antibody-BC42 Clone as an Alternative to the Current E6H4 for Use in the Female Genital Tract Pathological Diagnosis: Our Experience and a Review on p16ink4a Functional Significance, Role in Daily-Practice Diagnosis, Prognostic Potential, and Technical Pitfalls

**DOI:** 10.3390/diagnostics11040713

**Published:** 2021-04-16

**Authors:** Giuseppe Angelico, Angela Santoro, Frediano Inzani, Patrizia Straccia, Saveria Spadola, Damiano Arciuolo, Michele Valente, Nicoletta D’Alessandris, Roberta Benvenuto, Antonio Travaglino, Antonio Raffone, Gian Franco Zannoni

**Affiliations:** 1 Unità di Gineco-Patologia e Patologia Mammaria, Dipartimento Scienze della Salute della Donna, del Bambino e di Sanità Pubblica, Fondazione Policlinico Universitario A. Gemelli IRCCS, 00168 Roma, Italy; giuangel86@hotmail.it (G.A.); angela.santoro@policlinicogemelli.it (A.S.); frediano.inzani@policlinicogemelli.it (F.I.); patrizia.straccia@guest.policlinicogemelli.it (P.S.); saveriaspadola@hotmail.it (S.S.); damiano.arciuolo@policlinicogemelli.it (D.A.); dr.valente.m@gmail.com (M.V.); ndalessandris@gmail.com (N.D.); roberta.benvenuto@policlinicogemelli.it (R.B.); 2 Anatomic Pathology Unit, Department of Advanced Biomedical Sciences, University of Naples Federico II, 80125 Naples, Italy; antonio.travaglino.ap@gmail.com; 3 Gynecology and Obstetrics Unit, Department of Neuroscience, Reproductive Sciences and Dentistry, University of Naples Federico II, 80125 Naples, Italy; anton.raffone@gmail.com; 4 Istituto di Anatomia Patologica, Università Cattolica del Sacro Cuore, 00168 Roma, Italy

**Keywords:** p16, immunohistochemistry, squamous intraepithelial neoplasia, HPV, endocervical adenocarcinoma, endometrial carcinoma, mesenchymal tumors

## Abstract

Background: To date, useful diagnostic applications of p16 IHC have been documented in gynecological pathology both for HPV-related and non-HPV-related lesions. In the present article, we reported our experience with the novel anti-p16 INK4a antibody (clone BC42), whose expression was tested across all different gynecologic neoplasms; we also compared it to the traditional E6H4 clone. Moreover, we discussed and explored all the diagnostic applications of p16 IHC in gynecologic pathology. Methods: Consultation cases covering a 5-year period (2016–2020) regarding gynecological neoplastic and non-neoplastic lesions in which immunohistochemistry for p16, clone E6H4 was originally performed, were retrospectively retrieved from the files of our institution. Immunohistochemical staining for p16ink4a (BC42) [Biocare Medical group-Paceco USA; Bioptica Milan] and p16ink4a (E6H4) [Ventana Medical Systems-Arizona USA; Roche] was performed by using the Ventana automated immunostainer (Ventana Medical Systems, Tucson, AZ, USA). The immunostaining pattern was defined as negative, focal/patchy, or diffuse. Results: A total of 196 cases, represented by 36 high-grade SIL/CIN3 of the uterine cervix, 30 cervical adenocarcinomas, 22 cervical squamous cell carcinoma, 70 endometrial carcinomas, 25 high grade serous ovarian carcinomas, 6 uterine adenomatoid tumors, and 10 uterine leiomyosarcomas were included in this study. Results showed concordant staining quality of both clones on all tested neoplastic tissues. Conclusions: The novel anti-p16 antibody (BC42 clone) appeared as an alternative to the current E6H4 for use in gynecological neoplasms, offering similar levels of positivity and equally reliable staining results.

## 1. Introduction

p16 (INK4a) is a tumor suppressor protein encoded by the CDKN2A gene, located on chromosome 9p21 [1,2]. It acts as a cyclin-dependent kinase-4 inhibitor that is expressed in normal tissues and solid tumors. Its main function is to slow the progression of the cell cycle from the G1 to the S phase, acting as a tumor suppressor [1,2]. In detail, it binds to CDK4/6 and maintains the Rb gene product (pRb) in its hypo-phosphorylated state, which in turn binds to E2F transcription factor and prevents cell cycle progression [1,2].

Deletions in the CDKN2A gene can result in insufficient or non-functional p16 activity, thus accelerating the cell cycle and resulting in many types of cancer [1,2]. In most non-HPV-related tumors, including breast, pancreas, colon carcinomas, melanomas, and head and neck carcinomas related to smoking, the p16 function is lost by gene deletions, mutations, or epigenetic silencing. Hence, p16 IHC is usually negative in these tumors [3].

P16 expression pattern in normal human tissues varies with age; in fact, during childhood, it is expressed only in the thymus, while in adults, p16 expression is encountered in the endometrium, breast, gastric antral cells, esophageal squamous epithelium, salivary glands, and some neuroendocrine cells [3].

In neoplastic tissues, p16 positivity has been reported in dermatofibrosarcoma protuberans, gastric cancer, Hodgkin and non-Hodgkin lymphomas, neuroendocrine tumors, pulmonary carcinomas (both squamous and adenocarcinoma histotypes), and head and neck cancers [4,5,6].

To date, immunohistochemistry for p16ink4a is widely used as a surrogate marker for high-risk human papillomavirus (hrHPV) infection in formalin fixed-paraffin embedded (FFPE) tissues [3,5,7]. Therefore, its main diagnostic applications have been in the field of squamous lesions of the lower anogenital tract as well as squamous cell carcinomas of the head and neck and uterine cervix. In fact, in all HPV-related preneoplastic and neoplastic lesions, integration of the virus into the host cell genome leads to the production of E6 and E7 viral oncoproteins. E6 protein degrades p53, thus preventing apoptosis, while E7 protein inactivates pRb, preventing it from binding to the E2F transcription factor [3,8]. As a result, an increased expression of p16 in both the nucleus and cytoplasm can be detected by IHC.

Moreover, useful diagnostic applications of p16 IHC have been documented in gynecological pathology both for HPV-related and non-HPV-related lesions [3,5]. 

In the present article, after a state of the art regarding p16 diagnostic applications in gynecological pathology, we have reported our experience with a novel antibody: p16ink4a (BC42) [Biocare Medical group-Paceco USA; Bioptica Milan], and its expression was tested across all different gynecologic neoplasms and compared to the traditional E6H4 clone (Ventana Medical Systems-Arizona USA; Roche). 

## 2. Applications of p16 in Daily-Practice Gynecologic Pathology

### 2.1. Vulva

Normal vulvar squamous epithelium and squamous cell hyperplasia lack p16 immunostaining, while some authors reported a focal and heterogeneous expression in lichen sclerosus [9].

Regarding vulvar intraepithelial neoplasia (VIN), two main pathogenetic types are recognized: (1) Classic VIN, accounting for approximately 90% of cases, (2) differentiated (simplex) VIN, accounting for the remaining 10%. While classic VIN is related to HPV infection, differentiated VIN is not linked to HPV and is commonly associated with lichen sclerosus [10,11]. Therefore, p16 immunohistochemistry represents a reliable tool in the distinction between classic VIN (HPV-related, p16-positive) and differentiated VIN (p16 negative). On the other hand, p53 immunohistochemical expression is diffusely positive in differentiated VIN, and negative in the classic variant. Therefore, the combination of p16 and p53 antibodies is of great help in the distinction of these two pathogenetic pathways [5,10,11].

Moreover, the staining pattern of p16 is helpful in the distinction of the two main categories of classic VIN: Low-grade SIL (VIN 1) and High-grade SIL (VIN 2/3). In fact, a continuous strong nuclear or nuclear and cytoplasmic staining of the basal cell layer, which extends upward involving at least one-third of the epithelial thickness (block staining), is highly suggestive of precancerous high-grade SIL (VIN 2/3). On the other hand, p16 staining in VIN1 is usually limited to the basal cell layer [3,5,7,10,11].

Invasive vulvar neoplasms follow the same p16 staining pattern of their precursor lesions; in fact, HPV-related invasive squamous cell carcinomas are p16-positive, while non-HPV-related invasive tumors are mainly p16-negative [3,5]. An exception is represented by basal cell carcinomas, which may be p16-positive; therefore, its distinction from basaloid variant of squamous carcinoma cannot be based on p16 IHC [3,5]. Moreover, focal,” patchy” pattern of p16 staining has also been observed in vulvar Paget disease both in non-invasive and invasive forms [12].

### 2.2. Cervical Squamous Lesions

p16 expression in the squamous cervical epithelium is encountered in dysplastic, HPV related lesions: Low grade squamous intraepithelial lesion (L-SIL, CIN1) and high grade squamous intraepithelial lesion (H-SIL, CIN2-3); on the other hand, p16 is usually absent in normal squamous epithelium as well as in inflammatory conditions and in the metaplastic squamous epithelium [3,5,13,14,15]. 

L-SIL lesions display a nuclear and cytoplasmic positivity for p16 limited to the lower third of squamous epithelium; however, different staining patterns have been reported according to HPV type of infection: Intermediate and high-risk HPV-related L-SIL usually display strong and continuous p16 expression while low risk HPV-related L-SIL exhibits a less specific weak or focal p16 staining [3,5,13,14,15,16]. Moreover, several studies showed an association between p16 and H-SIL outcome: It seems that p16 positive CIN1 lesions are more likely to progress to CIN2-3, while only a minority of p16 negative CIN1 progress to high-grade dysplastic forms [5,16,17].

The most useful p16 application in cervical pathology is in the diagnosis of H-SIL. In fact, intense continuous p16 staining within the upper two thirds of the squamous epithelium is highly suggestive of the presence of a high-grade dysplastic lesion, although a small number of H-SILs can show no staining by p16 immunohistochemistry [3,5,18]. P16 use is recommended, especially in small cervical biopsies, to reduce interobserver variability derived from morphological evaluation alone [19]. Moreover, morphological mimickers of H-SIL, including atrophy, immature squamous metaplasia, and transitional metaplasia, can be easily ruled out based on their p16 negativity [5,20,21].

### 2.3. Glandular Lesions of Uterine Cervix and Endometrium

Endocervical glandular preneoplastic and neoplastic lesions related to HPV infection exhibit p16 positivity, which is not encountered in normal endocervical glands [5,22]. 

Diffuse p16 positivity is nearly always encountered in endocervical adenocarcinoma in situ (AIS), (given its association with high-risk HPV) and is of great diagnostic utility in the differential diagnosis with benign glandular lesions such as microglandular hyperplasia (MGH), tuboendometrial metaplasia (TEM), and endometriosis [5,22,23]. MGH is reported to be p16 negative, while TEM and endometriosis may exhibit p16 positivity, which is always focal and patchy in comparison to the diffuse staining pattern observed in AIS [5,22]. P16 expression is also encountered in invasive endocervical adenocarcinoma (including small cell neuroendocrine histotype), however, it is not limited to HPV-related histotypes [5,22,23,24,25]. In fact, up to one-third of non-HPV-related, gastric type adenocarcinomas have been shown to express p16, representing thus a diagnostic pitfall [25,26].

Another diagnostic field in which p16 IHC plays a key role is in the distinction between endocervical and endometrial adenocarcinoma since the precise site of origin (endometrium vs. cervical canal) has important prognostic and therapeutic implications [5,23,27,28,29]. Fortunately, since endometrial adenocarcinoma is non-HPV-related, it usually exhibits a focal and patchy type of staining (with the exception of areas of squamous metaplasia), which contrasts with the diffuse expression encountered in endocervical cancer. The only exception is represented by high-grade serous histotype, which is diffusely p16 positive despite harboring no relationships with HPV [5,23,27,28,29].

However, to better clarify the endometrial versus endocervical origin of neoplasms, p16 must be integrated with broader immunohistochemical panels, which include estrogen and progesterone receptors as well as vimentin, monoclonal carcinoembryonic antigen, and mismatch repair proteins [5,23,27,28,29,30,31].

Finally, p16 has been recently proposed as a diagnostic marker to differentiate atypical polypoid adenomyoma (APA) from myoinvasive endometrioid carcinoma; indeed, p16 often shows diffuse positivity in the fibromyomatous stroma of APA and negativity or focal positivity in the stroma of myoinvasive carcinoma [32,33,34].

### 2.4. Mesenchymal Tumors of the Female Genital Tract

Despite the fact that p16 has limited diagnostic utility in mesenchymal tumors, interesting data have been published regarding its expression in uterine smooth muscle tumors. Several studies reported significant differences in p16 staining intensity and frequency between leiomyomas, STUMPs, and leiomyosarcomas. In fact, diffuse and strong staining for p16 is usually encountered in leiomyosarcomas and less frequently in STUMPS, while most leiomyomas are reported to be p16 negative [5,35,36,37]. Moreover, Ip et al. demonstrated that p16 expression in STUMPs, may be related to increased risk of recurrence [36]. 

Uterine tumors resembling ovarian sex cord tumors (UTROSCT) represent rare mesenchymal tumors with sex cord differentiation where sporadic reports have highlighted p16 positive staining [38,39]. Finally, p16 can also be expressed by adenomatoid tumors of the female genital tract. However, limited data are still available on the pathogenetic role of p16 in these rare subsets of mesenchymal neoplasms [40].

### 2.5. Ovary

p16 IHC has limited diagnostic applications in ovarian neoplasms. In general, p16 is negative or weekly positive in most ovarian tumors, including benign and borderline serous tumors, endometrioid, mucinous, clear cell tumors, as well as sex cord and germ cell neoplasms [5,40,41,42,43,44]. Therefore, one common diagnostic use of p16 is in the differential diagnosis of synchronous or metachronous cervical and ovarian adenocarcinomas. In this scenario, strong and diffuse p16 expression is suggestive of a cervical origin [5,41,42,43,44,45].

The only primitive ovarian tumor exhibiting diffuse and strong p16 expression is high-grade serous ovarian carcinoma. This finding contrasts with the low and weak staining encountered in low grade, borderline, and serous neoplasms [5,41,42,43,44,45]. In this way, p16 is thought to play a pathogenetic role in the development of high-grade serous carcinoma, along with the other well-known genetic pathways involving TP53, PPP2R1A, CTNNB1, and PIK3CA mutations [46,47].

## 3. Materials and Methods

### 3.1. Ethic Statement and Patient Selection 

We have compared the new anti-p16 INK4a antibody (BC42 clone, Biocare Medical group-Paceco USA; Bioptica Milan) to clone E6H4, in terms of staining results on various neoplastic tissues of the female genital tract in order to assess its diagnostic reliability in daily diagnostic practice.

All consultation cases covering a 5-year period (2016–2020) regarding gynecological neoplastic and non-neoplastic lesions in which immunohistochemistry for p16, clone E6H4, was originally performed, were retrospectively retrieved from the files of our institution.

### 3.2. Pathological Assessment and Immunohistochemistry (IHC) 

Pathology reports, hematoxylin-and-eosin (H&E), and immunohistochemical stained slides were reviewed by 2 expert pathologists (G.F.Z., F.I.), in order to confirm the initial histological diagnoses. Sections (~4 μm) of formalin-fixed paraffin-embedded tissues were deparaffinized, rehydrated through a series of alcohol/water solutions, followed by blocking of endogenous peroxidases with a 3% hydrogen peroxide solution. Tissues were subjected to heat-induced antigen retrieval using a modified citrate buffer in a pressure cooker (Decloaking Chamber; Biocare Medical, 60 Berry Dr, Pacheco, CA 94553, USA) and were heated to 110 °C for 15 min. Both p16 antibodies (BC42, Bioptica Milan, Biocare Medical group-Paceco USA; and E6H4, Roche diagnostics, Basel, Switzerland.) were applied to target tissues for 60 min. Detection of the p16 antibody was accomplished using a MACH 4 Universal HRP-Polymer detection system (Biocare Medical, 60 Berry Dr, Pacheco, CA 94553, USA). In a final detection step, 3,3′-diaminobenzidine (DAB) was applied for visualization. Slides were briefly counterstained in a modified Mayer’s hematoxylin. Immunohistochemical staining was performed by using the Ventana automated immunostainer (Ventana Medical Systems, Tucson, AZ, USA). Diaminobenzidine was used as the chromogen, and sections were counterstained in Harris’ hematoxylin. Positive controls were included in each immunostaining run. These comprised a cervical squamous carcinoma with diffuse, strong positivity with p16. 

p16 immunostaining pattern was defined as negative, focal/patchy, or diffuse. A focal pattern consisted of patchy and discontinuous staining with mainly cytoplasmic localization and minor nuclear staining. A diffuse pattern was defined as diffuse and strong nuclear and cytoplasmic staining.

## 4. Results

A total of 196 cases, represented by 36 high-grade SIL/CIN2-3 of the uterine cervix, 30 cervical adenocarcinomas, 22 cervical squamous cell carcinoma, 70 endometrial carcinomas (40 endometrioid and 30 serous), 25 high grade serous ovarian carcinomas, 6 uterine adenomatoid tumors, and 10 uterine leiomyosarcomas were included in this study.

Results showed concordant staining quality of both clones on all neoplastic tissues tested. All tested cases and comparative immunohistochemical results are reported in Table 1.

### 4.1. Neoplastic Lesions of the Uterine Cervix

**Figure 1:**
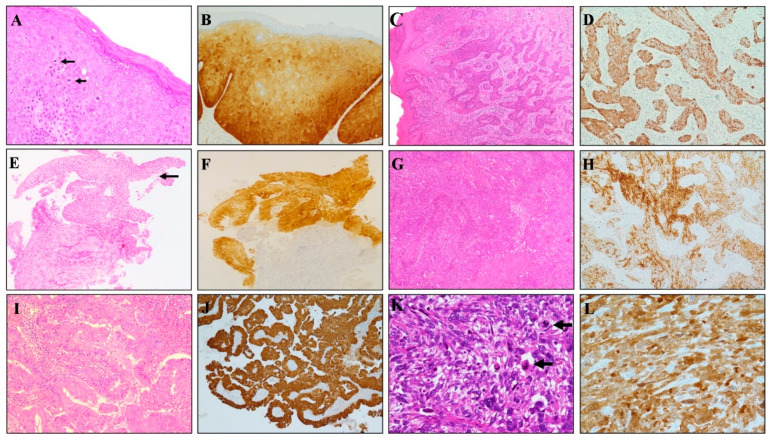
Diagnostic applications of p16 INK4a (BC42 clone) in different gynecological tumors. (**A**) Vulvar intraepithelial neoplasia (high-grade SIL/VIN 3) is characterized by full-thickness nuclear atypia and increased mitotic activity (arrows). (**B**) A continuous strong nuclear and cytoplasmic p16 staining across all epithelial layers is observed. (**C**) HPV-related invasive squamous cell carcinoma of the vulva showing diffuse and strong p16 (BC42) staining (**D**). (**E**) Cervical bioptic specimen showing high-grade dysplasia of the surface epithelium (arrow) confirmed by the strong nuclear and cytoplasmic staining for p16 (**F**). (**G**) HPV-related invasive squamous cell carcinoma of the uterine cervix showing diffuse and strong p16 (BC42) staining (**H**). (**I**) HPV-related invasive adenocarcinoma of the uterine cervix showing diffuse and strong p16 (BC42) staining (**J**). (**K**) Uterine spindle cell leiomyosarcoma is characterized by high-grade nuclear atypia and increased mitotic activity (arrows). (**L**) A strong nuclear and cytoplasmic p16 staining is observed.

Strong and diffuse staining pattern with both BC42 and E6H4 clones was observed in 35/36 cervical high-grade SIL cases (Figure 1); in detail, diffuse p16 positivity with both clones was observed in 18/18 CIN3 and 17/18 CIN2 lesions, with only one CIN2 showing negative staining for both p16 antibodies.

Strong and diffuse p16 staining was also encountered in all usual-type endocervical adenocarcinomas (30 cases) and cervical squamous cell carcinomas (22 cases). 

### 4.2. Neoplastic Lesions of the Uterine Corpus

**Figure 2:**
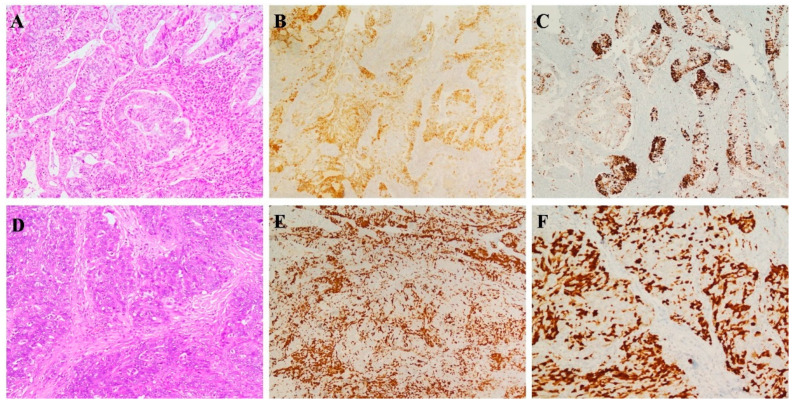
p16 INK4a comparative immunostains with E6H4 and BC42 clone tumors. (**A**) Moderately differentiated endometrioid endometrial adenocarcinoma showing a similar focal/patchy staining pattern for p16 with E6H4 (**B**) and BC42 (**C**) clones. (**D**) High-grade ovarian serous carcinoma demonstrating similar distribution and staining intensity with both E6H4 (**E**) and BC42 (**F**) clones.

Regarding p16 expression in endometrial tumors, focal/patchy pattern of p16 staining, was detected with both clones in 32/40 (80%) tested in endometrial endometrioid carcinomas (Figure 1 and Figure 2); the remaining endometrioid cases (8/40, 20%) exhibited 100% positivity; a strong and diffuse staining pattern was also observed in 30/30 (100%) uterine serous adenocarcinoma. Both clones evidenced diffuse marker staining in all tested uterine adenomatoid tumors (6/6 cases). Among 10 uterine leiomyosarcoma, strong and diffuse p16 positivity with BC42 and E6H4 clones was observed in 4 and 5 cases, respectively, with the remaining 2 cases showing a focal/zonal/heterogeneous pattern.

### 4.3. p16 Expression in High-Grade Ovarian Cancer

Ovarian high-grade serous carcinomas also showed identical staining results with both clones. In detail, strong and diffuse p16 positivity with BC42 and E6H4 clones was observed in 21/25 and 20/25 cases, respectively, with the remaining cases showing a “patchy” pattern of staining (Figure 1 and Figure 2).

## 5. Discussion

In recent years, p16 has been extensively investigated as a diagnostic aid in various scenarios in gynecologic pathology. Like with all markers, in each of these scenarios, p16 is neither 100% specific nor 100% sensitive for a given lesion. 

In the gyneco-pathological scenario, there are several areas where p16 is of undoubted value, usually in combination with other markers. These include: (i) The distinction of uVIN from dVIN in vulvar site; (ii) the identification of small high-grade CIN lesions, in particular at cauterized cervical resection margins; (iii) the separation of high grade CIN from benign mimics; (iv) the distinction between low grade and high grade CIN; (v) the distinction of cervical AIS from benign mimics; (vi) the identification of cervical small cell carcinoma; (vii) its potential prognostic role as predictor of HSIL outcome; (viii) the distinction of an endometrial and an endocervical adenocarcinoma; (ix) the differential diagnosis between uterine endometrioid adenocarcinomas and serous carcinoma (in particular in presence of glandular pattern); (x) the confirmation of a metastatic cervical adenocarcinoma in the ovary.

It is likely that as the full range of p16 immunoreactivity is further investigated, additional useful applications will be discovered. Specific areas worthy of future investigation include the value of p16 in the categorization of problematic uterine smooth muscle neoplasms, the differential expression of p16 in low-grade and high-grade ovarian serous carcinomas, and the role of p16 as a diagnostic marker of APA. 

Different tested monoclonal clones are commercially available, but data comparing protocols of use are lacking [48,49]. In routinary diagnosis, the most commonly used antibody is a mouse monoclonal antibody anti-p16 [E6H4] developed and commercially offered by ROCHE, Basel, Switzerland, with a Food and Drug Administration (FDA) approved version of the test in order to resolve or minimize issues related to laboratory standardization [50].

Recently, a novel anti-p16 antibody has emerged as an alternative to the current E6H4 for use in histopathological diagnosis. It has been obtained by immunizing Balb/C mice with a recombinant human p16 protein [49]. 

The Anti-p16 INK4a antibody [clone BC42] has been already tested on various normal (30 types) and neoplastic tissues (12 types), revealing equivalent exceptional staining quality, high levels of sensitivity and specificity compared to the current used clone E6H4, in particular in: Cervical adenocarcinoma (22 cases); cervical intraepithelial neoplasia (CIN) (24 cases); cervical squamous carcinoma (16 cases); head and neck cancer (12 cases); endometrial carcinoma (48 cases) and ovarian cancer (12 cases) [51].

Our study represents the first one testing staining quality and applicability of the new clone BC42 on a larger number of gynecological cases (196 cases), also including uterine leiomyosarcomas and adenomatoid tumors. Results showed high staining quality on all neoplastic tissues tested, similarly to the current used clone E6H4.

In particular, a strong and diffuse staining pattern with both BC42 and E6H4 clones was observed in 97.22% of H-SIL, in 100% of usual type cervical adenocarcinomas, cervical squamous cell carcinomas, and uterine adenomatoid tumors, and, respectively, in 84% vs. 80% ovarian high-grade serous carcinomas.

We must be aware that p16 staining, as well as any type of immunohistochemical staining, can be affected by:

- Pre-analytical variables: Specimen collection and handling, tissue fixation (uniformity, time, and type), and protocol of processing
- Analytical variables: Choice of immunohistochemistry protocol, reagent variability, antigen retrieval technique, and technician training/expertise
- Post-analytical variables: Evaluation of positive/negative controls, morphological correlations, diagnostic and prognostic significance, correlation with other data, interpretation, and reporting of results, and experience/expertise of the pathologist

The goal consists in ensuring reproducibility, obtaining high quality stained sections with minimal inter-observer variability in diagnostic report and in promoting inter-laboratory standardization, which are all synonyms of quality assurance, defined by the College of American Pathologists (CAP) as a ‘‘process of assuring that all pathology services involved in the delivery of patient care have been accomplished in a manner appropriate to maintain excellence’’ [50]. We retain that it can be achieved only by the application of automated systems (e.g., Ventana and Dako systems) [52,53].

## 6. Conclusions

P16 IHC is of crucial diagnostic importance in several fields of gynecopathology, and its area of application is further increasing.

The new anti-p16 antibody (BC42 clone) is emerging as an alternative to the current E6H4 for use in cancer pathological diagnosis, offering similar levels of positivity and equally reliable staining results.

We have to keep in mind that to achieve the excellence in a pathological diagnosis, concerted actions with regard to laboratory standardization, quality assurance, coupled with progressive automation of all procedures and technical/medical skills are needed for the proper utility of p16 IHC in daily practice.

## Figures and Tables

**Table 1:** BC42 and E6H4 comparison staining across all gynecological neoplasms tested.

Tissue	Total Cases	BC42+	% Positive	E6H4+	% Positive
H-SIL	36	35	97.22	35	97.22
-CIN3	18	18	100	18	100
*-CIN2*	18	17	94.44	17	94.44
Endocervical Usual type Adenocarcinoma	30	30	100	30	100
Cervical Squamous cell carcinoma	22	22	100	22	100
Endometrial Carcinoma	70	70	100	70	100
-endometrioid type	40	32	80 focal/patchy	32	80 focal/patchy
		8	20 diffuse	8	20 diffuse
-serous type	30	30	100	30	100
Ovarian High-grade Serous Carcinoma	25	21	84	20	80
Uterine Adenomatoid Tumor	6	6	100	6	100
Uterine Leyomiosarcoma	10	6	60	7	70
		4 diffuse		5 diffuse	
		2 focal		2 focal	

## Data Availability

All data are available upon reasonable request to corresponding author.
